# Supplementary material for: Resistance to TGFβ suppression and improved anti-tumor responses in CD8+ T cells lacking PTPN22
Source: Nat Commun. 2017 Nov 7;8:1343. doi: 10.1038/s41467-017-01427-1 (PMC5676842; doi:10.1038/s41467-017-01427-1)
Supplement: Supplementary file 1 — Supplementary Information [file 41467_2017_1427_MOESM1_ESM.pdf]

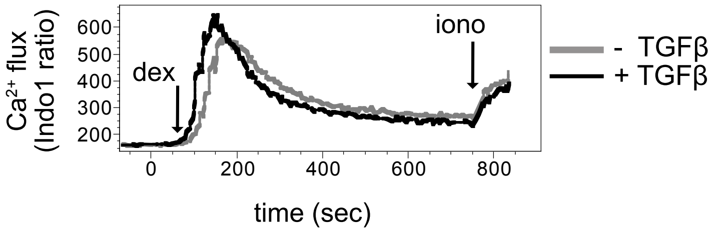

Supplementary Fig. 1. Calcium flux induced by multivalent peptide:MHC complexes is not suppressed by TGFβ.

OT1 cells loaded with Indo-1 AM dye were either pre-treated or not with 5ng/ml TGFβ for 5 mins prior to stimulation with N4-dextramer (dex) to stimulate a Ca^{2+} flux. Ionomycin (iono) was added as a positive control as indicated on graph. The Ca^{2+} flux was measured by flow cytometry and is representative of 3 independent experiments.

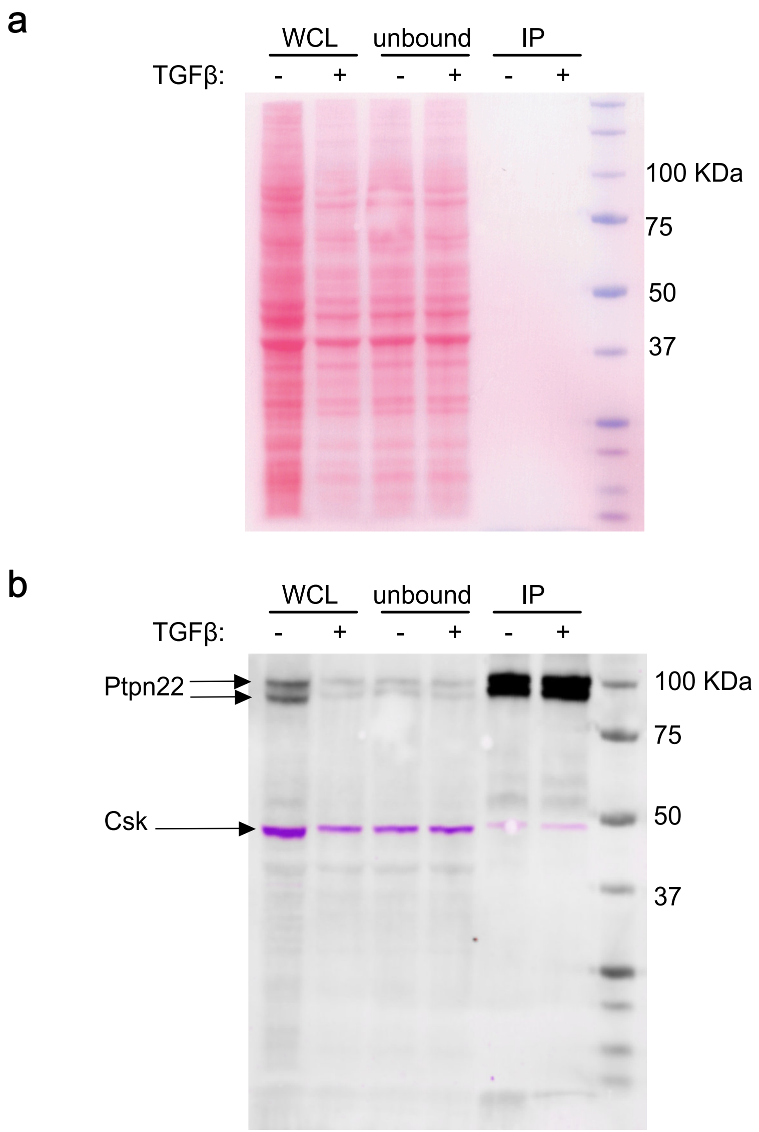

Supplementary Fig. 2. Uncropped gel images for western blot. (**a**) shows ponceau stain and (**b**) shows uncropped image corresponding to **Fig. 4f**. (WCL= whole cell lysate, IP = immunoprecipitation)
